# Simultaneous polyhydroxyalkanoates and rhamnolipids production by *Thermus thermophilus *HB8

**DOI:** 10.1186/2191-0855-1-17

**Published:** 2011-07-13

**Authors:** Anastasia A Pantazaki, Christos P Papaneophytou, Dimitra A Lambropoulou

**Affiliations:** 1 Laboratory of Biochemistry, Dept. of Chemistry, Aristotle University of Thessaloniki, 54124 Thessaloniki, Greece; 2 Environmental Pollution Control Laboratory, Dept. of Chemistry, Aristotle University of Thessaloniki, 54124 Thessaloniki, Greece

**Keywords:** polyhydroxyalkanoates (PHAs), rhamnolipids (RLs), phosphate limitation, *Thermus thermophilus *HB8

## Abstract

The ability of *Thermus thermophilus *HB8 to produce simultaneously two environmentally-friendly biodegradable products, polyhydroxyalkanoates (PHAs) and rhamnolipids (RLs), using either sodium gluconate or glucose as sole carbon source, was demonstrated. The utilization of sodium gluconate resulted in higher levels of PHAs and RLs production than when glucose was used as sole carbon source. The initial phosphate concentration (as PO_4_^3-^) influences both PHAs and RLs productions that were increased during cultivation time. PHAs accumulation was enhanced (> 300 mg/L) after 72 h of cultivation in an initial [PO_4_^3-^] of 25 mM, while RLs production (> 200 mg/L) was started after 35 h and continued until 72 h of cultivation, in a phosphate-limited medium containing initially 5 mM of [PO_4_^3-^]. In addition, the combine effect of initial [PO_4_^3-^] and cultivation time on biomass, PHAs and RLs production was evaluated from 2D contour plots. The results revealed that low initial phosphate concentrations (up to 5 mM) and long incubation time (72 h) promoted RLs biosynthesis while higher initial phosphate concentrations (up to 25 mM) where favorable for biomass and PHAs production. The molecular composition of the produced bio-products was identified. The accumulated PHAs were co-polymers which mainly consisted of 3-hydroxydecanoate (3HD) as resulted by gas chromatography (GC) analysis. The secreted RLs were extracted and their total mixture contained both mono- and di- RLs identified by thin-layer chromatography (TLC). Moreover, the molecular composition of the produced RLs characterized in details by LC-MS analysis showed a plethora of diversity including mono-, and di-RLs, di-rhamno-monolipidic congeners differing in the length of the lipidic chain, which additionally were found to be saturated or unsaturated in some cases.

## Introduction

A wide variety of microorganisms accumulate polyhydroxyalkanoic acids (PHAs), mainly polyhydroxybutyrate (PHB), as metabolic storage materials, which are deposited as intracellular water-insoluble inclusions (Griebel et al. 1968; Rehm and Steinbüchel 1999). Most fluorescent *Pseudomonads *strains belonging to rRNA homology group I, are able to synthesize and accumulate large amounts of PHAs consisting of various 3-hydroxy fatty acids with carbon chain lengths ranging from 6 to 14 carbon atoms (medium chain length-mcl) as carbon and energy storage compounds (Huisman et al. 1989). The composition of PHAs depends on the PHA synthases (polymerases) (PhaC), the key enzymes for PHA biosynthesis, the carbon source and the metabolic routes involved (Rehm 2003; Rehm and Steinbüchel 1999). Purified mcl-PHA synthases, from *P. aeruginosa *exhibit *in vitro *enzyme activity with (R)-3-hydroxydecanoyl-CoA as substrate (Amara and Rehm 2003; Qi et al. 2000; Ren et al. 2000). β-Oxidation is the main pathway for mcl-PHA biosynthesis when fatty acids are used as carbon sources, while de novo biosynthesis of fatty acids is the main route during growth on carbon sources which are metabolized to acetyl coenzyme A (acetyl-CoA), such as gluconate, acetate, or ethanol (Rehm et al. 2001). Besides the intracellular accumulation of PHAs, *P. aeruginosa *is capable to produce various exo-products, such as rhamnolipids (RLs), which are expressed in the onset of the stationary phase (Déziel et al. 1996).

RLs are glycolipidic bio-surfactants, which reduce water surface tension and emulsify oil. The RLs produced by *P. aeruginosa *in liquid cultures are mainly rhamnosyl-β-hydroxydecanoyl-β-hydroxydecanoate (mono-RL) and rhamnosyl-rhamnosyl-β-hydroxydecanoyl-β-hydroxydecanoate (di-RLs) (Ochsner and Reiser 1995). RL biosynthesis proceeds through transfer of two rhamnose moieties from TDP-L-rhamnose (Maier and Soberón-Chávez 2000). For the synthesis of mono-RL, the enzyme rhamnosyl-transferase 1 (Rt 1) catalyses the rhamnose transfer to β-hydroxydecanoyl-β-hydroxydecanoate, while rhamnosyl-transferase 2 (Rt 2) synthesizes di-RL from TDP-L-rhamnose and mono-RL. Genes for biosynthesis, regulation and induction of the Rt 1 enzyme are organized in tandem in the rhlABRI gene cluster (Ochsner and Reiser 1995). The gene rhlC encoding the Rt 2 enzyme has been described (Rahim et al. 2001), and is homologous to rhamnosyl-transferases involved in lipopolysaccharide biosynthesis. Some evidence was recently provided that RhlA is involved in synthesis of 3-(3-hydroxyalkanoyloxy)alkanoic acids (HAAs) (Déziel et al. 2003); however, it still remains unclear whether the PHA synthase is capable of catalyzing the synthesis of HAAs, which has been previously postulated (Campos-Garcia et al. 1998; Déziel et al. 2003; Rehm et al. 2001).

The expression of *phaG *gene, encoding transacylase from *P. putida *in mutants indicated that PhaG catalyzes diversion of intermediates of fatty acid de novo biosynthesis towards PHA biosynthesis, and in the transacylase-mediated PHA biosynthesis route from gluconate, PhaG is the only linking enzyme between fatty acid de novo biosynthesis and PHA biosynthesis. In addition, expression of the *β*-ketoacyl reductase gene *rhlG *from *P. aeruginosa *in mutants revealed that RhlG catalyzes diversion towards RL biosynthesis (Campos-Garcia et al. 1998). RhlG is thought to catalyze the NADPH-dependent reduction of β-ketodecanoyl-ACP, which is an intermediate of fatty acid de novo biosynthesis, resulting in β-hydroxydecanoyl-ACP, a putative precursor for RL biosynthesis. The proposed pathways for mcl-PHA and RLs biosynthesis suggested that both biosynthesis pathways are competitive (Rehm et al. 2001).

While the opportunistic pathogen *P. aeruginosa *has traditionally been considered the primary PHAs and RLs-producing microorganism, however it appears that the ability to produce PHAs and especially RLs (Abdel-Mawgoud et al. 2010) is in fact restricted to a limited number of bacterial species. Since there are a plethora of applications for both metabolites PHAs (Zinn et al. 2001; Valappil et al. 2006) and RLs (Abdel-Mawgoud et al. 2010) a low cost production would be very challenging. A novel approach for reducing their production costs was also reported for the simultaneous production of the intracellular PHAs and the extracellular RLs (Hori et al. 2002). PHAs production was reported using the remaining oil from RL production (Füchtenbusch et al. 2000). This approach was adopted to reduce PHA production cost by using the remaining carbon source for RL production. Palm oil, one of the typical plant oils, was also used as the sole carbon source for the simultaneous production of both products (Marsudi et al. 2008).

Bio-surfactants are produced by certain bacteria, yeasts, and filamentous fungi during cultivation on various carbon sources, in particular during growth on hydrophobic substances such as hydrocarbons leading to the assumption that biosurfactants serve to emulsify the hydrocarbons in the growth medium thus facilitating their uptake. A correlation between surfactant production and growth on water-insoluble substrates was shown (Itoh and Suzuki 1972). RLs are usually produced by *P. aeruginosa *also in media containing water-soluble carbon sources such as glucose, glycerol, mannitol, and ethanol as a carbon source, particularly, when the cells become limited for nitrogen (Guerra-Santos et al. 1986; Mulligan and Gibbs 1993; Robert et al. 1989; Sim et al. 1997; Wagner et al. 1983) indicating that they may serve other roles besides being involved in solubilizing hydrophobic substrates; nevertheless, the final surfactant concentration was lower than that obtained when *n*-alkanes and vegetable oils were the carbon substrates (Syldatk et al. 1985; Robert et al. 1989). The carbon source influences bio-surfactant synthesis by either induction or repression. An induction of glycolipid production by *P. aeruginosa *SB30, when *n*-alkanes were added to the medium was reported (Chakrabarty 1985). Catabolic repression of bio-surfactant synthesis by glucose, acetate, and tricarboxylic acids was also observed (Hauser and Karnovsky 1958, 1954). When *P. aeruginosa *UG2 was grown on hydrophobic substrates such as corn oil, lard, and long chain alcohols, production was around 100-165 mg of rhamnolipid/g substrate, whereas when hydrophilic substrates such as glucose and succinic acid were used, only 12-36 mg/g substrate was obtained (Mata-Sandoval et al. 1999).

Polyhydroxyalkanoates (PHAs) are intracellular storage compounds of carbon and energy that are produced by many bacteria in the form of inclusion bodies (Anderson and Dawes 1990). PHAs have attracted commercial biotechnological interest because of their biodegradability and biocompatibility (Reddy et al. 2003).

PHA biosynthesis by the thermophilic bacterium *T. thermophilus *HB8 has been previously reported (Pantazaki et al. 2003) while the soluble PHA synthase (Pantazaki et al. 2003), and a *β*-ketoacyl-CoA thiolase (Pantazaki et al. 2005), were purified from sodium gluconate-grown cells. In addition, PHAs production by *T. thermophilus *HB8 using whey as sole carbon source was also reported (Pantazaki et al. 2009). Recently, it was also demonstrated that *T. thermophilus *secreted RLs, when grown in the presence of insoluble substrates such as sunflower seed oil or oleic acid. The produced RLs have been identified by several methods e.g the orcinol method, Thin Layer Chromatography (TLC) and Attenuated Total Reflection Infrared (ATR-FTIR) spectroscopy and LC-(ESI)-MS (Pantazaki et al. 2010).

In this study we report the simultaneous production of both metabolites PHAs accumulated intra-cellularly and RLs secreted extra-cellularly by employing *T. thermophilus *HB8, as an approach to turn to advantage from the carbon source used extensively for the production of high added value bio-products from wastes aiming to obtaining the reduction of production cost. In this concept, this study specifically focused in finding the optimum growth condition by examining the combine effect of cultivation time and initial [PO_4_^3-^] on the simultaneous production of these metabolites to increase their production. The identification of the molecular composition of the produced PHAs and bio-surfactants RLs was also evaluated by GC chromatography and thin-layer chromatography (TLC)/LC-MS analysis respectively.

## Materials and methods

### Bacterial strain and growth

*T. thermophilus *HB8 (DSM 579) was grown in a rich medium (DSMZ-74) containing per liter: 8 g tryptone, 4 g yeast extract, and 2 g NaCl. For PHAs and RLs production, cultivation was carried out in 2 L Erlenmeyer flasks containing 700 mL mineral salt medium (MSM) containing per liter: 1 mM KH_2_PO_4, _25 mM Na_2_HPO_4_.12 H_2_O, 0.05 g NH_4_Cl,10 g NaCl, 15 mg CaCl_2_, 123 mg MgSO_4_.7H_2_O, 6 mL of a mineral solution (Pantazaki et al. 2003). *T. thermophilus *HB8 cultures were grown in the presence of the selected carbon source at 75°C. Glucose (2% w/v) or sodium gluconate (1.5% w/v) were used as sole carbon sources.

To investigate the influence of the initial [PO_4_^3-^] on both PHAs and RLs production, *T. thermophilus *was grown in MSM containing per liter: 2 g NH_4_Cl, 10 g NaCl, 15 mg CaCl_2_, 123 mg MgSO_4_.7H_2_O, 6 mL of a mineral solution (Pantazaki et al. 2003) and one of the following initial phosphate concentrations: 0.5, 5, 10, 25 and 50 mM. Phosphate concentration was adjusted by the addition of the proper amount of a 0.5 M phosphate buffer, pH 7.2. Sodium gluconate was used as sole carbon source at a concentration of 1.5 (% w/v).

### Biomass and PHA content determination in cells

During cultivation aliquots of 100 mL were removed from each of the cultures, at various time intervals, and were centrifuged at 6,800 × g for 15 min to separate the biomass from the supernatants. Biomass was used for the determination of PHAs content in the cells, while the supernatants obtained were destined for RLs determination. Cell concentration, defined as cell dry weight (CDW) per liter of culture broth in each time interval during cultivation, was determined by filtrating 10 mL of culture broth using tarred membranes filters (Millipore, 0.45 μm filters), washing cell pellets, drying at 80°C for 48 h, and weighing dry cells as previously reported (Pantazaki et al. 2009). The content of polyester in the cells was calculated as the ratio of weight of extracted PHAs to the cell dry weight from which PHAs were extracted.

### PHAs characterization

The monomer composition of the accumulated PHAs as well their content in the cells, were determined by gas chromatography (GC) as previously reported (Braunegg et al. 1978). For this lyophilized *T. thermophilus *HB8 cells (10 mg) were subjected to methanolysis in the presence of 15% (v/v) sulfuric acid (Braunegg et al. 1978), for extraction of polyester and its transformation in the corresponded 3-hydroxyalkanoic methyl esters. Gas chromatography was performed with a Varian 3300 gas chromatograph equipped with an OV-351 capillary column (15 m × 0.53 mm; OHIO VALLEY Capillaries, 115 Industry Road, Marietta, OH 45750, U.S.A.) and a flame ionization detector. Two microliters of the organic phase was injected in the column. Nitrogen (1 mL/min) was used as the carrier gas. The temperature of the injector and detector were 200 and 230°C, respectively. A temperature program was used for efficient separation of the esters [60°C for 1 min, temperature increase 8°C/min until 160°C, temperature increase 25°C/min to 230°C (230°C for 1 min)]. Under these conditions, the retention times (in minutes) of the different 3-hydroxyalkanoic acid methyl ester standards were as follows: C4, 3.18; C5, 4.16; C6, 5.24; C7, 7.11; C8, 7.95; C9, 9.46; C10, 10.53; C11: 11.9; C12, 13.2 (CX represents the 3-hydroxyalkanoic acid methyl ester with a chain length of X carbon atoms).

### Extraction and analysis of RLs

Produced RLs were extracted from the culture supernatants, as previously described (Hori et al. 2002) and quantified by the colorimetric orcinol method (Koch et al. 1991). Three independent samples were extracted for each cultivation time to monitor the reproducibility of the rhamnolipids extraction procedure. RLs concentration was calculated by a coefficient of 3.4, obtained from the correlation of pure rhamonlipids/rhamnose (1.0 mg of rhamnose corresponds approximately to 3.4 mg of rhamnolipids) (Pearson et al. 1997). RLs were analyzed by thin-layer chromatography (TLC) on silica gel plates (Kieselgel 60/Kieselgur F254; Merck, Darmstadt, Germany). Samples were spotted on the plates and separated using a mobile phase consisting of 80% (v/v) chloroform, 18% (v/v) methanol and 2% (v/v) acetic acid. TLC plates were sprayed with a thymol spray reagent [0.5 g thymol in EtOH-conc. H_2_SO_4 _(95:5, v/v)]; after spraying plates were heated for 20 min at 100 to 120°C for 20 min, and the RLs appeared as pink spots (Schenk et al. 1995).

### Determination of glucose and phosphate concentration

Remaining glucose concentration in culture media was measured by an enzymatic colorimetric method using a commercially available kit (Sigma GAG020). Glucose is oxidized to gluconic acid and hydrogen peroxide by glucose oxidase. Hydrogen peroxide reacts with *o*-dianisidine in the presence of peroxidase to form a colored product. Oxidized *o*-dianisidine reacts with sulfuric acid to form a more stable colored product. The intensity of the pink color measured at 540 nm is proportional to the original glucose concentration.

Determination of inorganic phosphate (as PO_4_^3-^) was performed with the conventional colorimetric method (Fiske and SubbaRow 1925).

### LC-MS analysis of RLs

ESI-MS in both negative and positive mode was performed using an Agilent G2455A ion trap mass spectrometer equipped with Agilent software. The collected samples of RLs were dissolved in MeOH/water (0.1% Formic acid) and infused in the ESI source with flow rate of 100 μL/min. ESI-MS spectra (positive or negative mode) were acquired from m/z 50-1200 for 2.5 min. Operating conditions were as follows: accumulation time, 300 ms; dry temperature, 350 C; capillary voltage, 3500 V; nebulizer, 30 psi; dry gas, helium at 8 L/min.

## Results

### Effect of carbon source on simultaneous PHAs and RLs production by *T. thermophilus *HB8

The ability of *T. thermophilus *to produce simultaneously PHAs and RLs was initially tested in MSM containing either glucose (2% w/v) or sodium gluconate (1.5% w/v) as sole carbon source while the initial phosphate concentration was adjusted at approximately 36 mM (see Material and Methods section). Glucose was selected since is the most commonly used sugar substrate in microbial cultivations, while sodium gluconate derived from glucose, its metabolic precursor, in which glucose is transformed via Entner-Doudoroff pathway by glucose dehydrogenase with the NAD(P)^+ ^reduction. In addition, the utilization of glucose by *Pseudomonas *for the RLs (Guerra-Santos et al. 1986) or PHAs (Huijberts et al. 1992) production has been previously reported. Moreover, glucose is an attractive carbon source because it can be obtained by hydrolyzing cheap raw materials such corn starch (Lee 1996) or organic waste from the food industry (Rusendi and Sheppard 1995).

**Figure 1:**
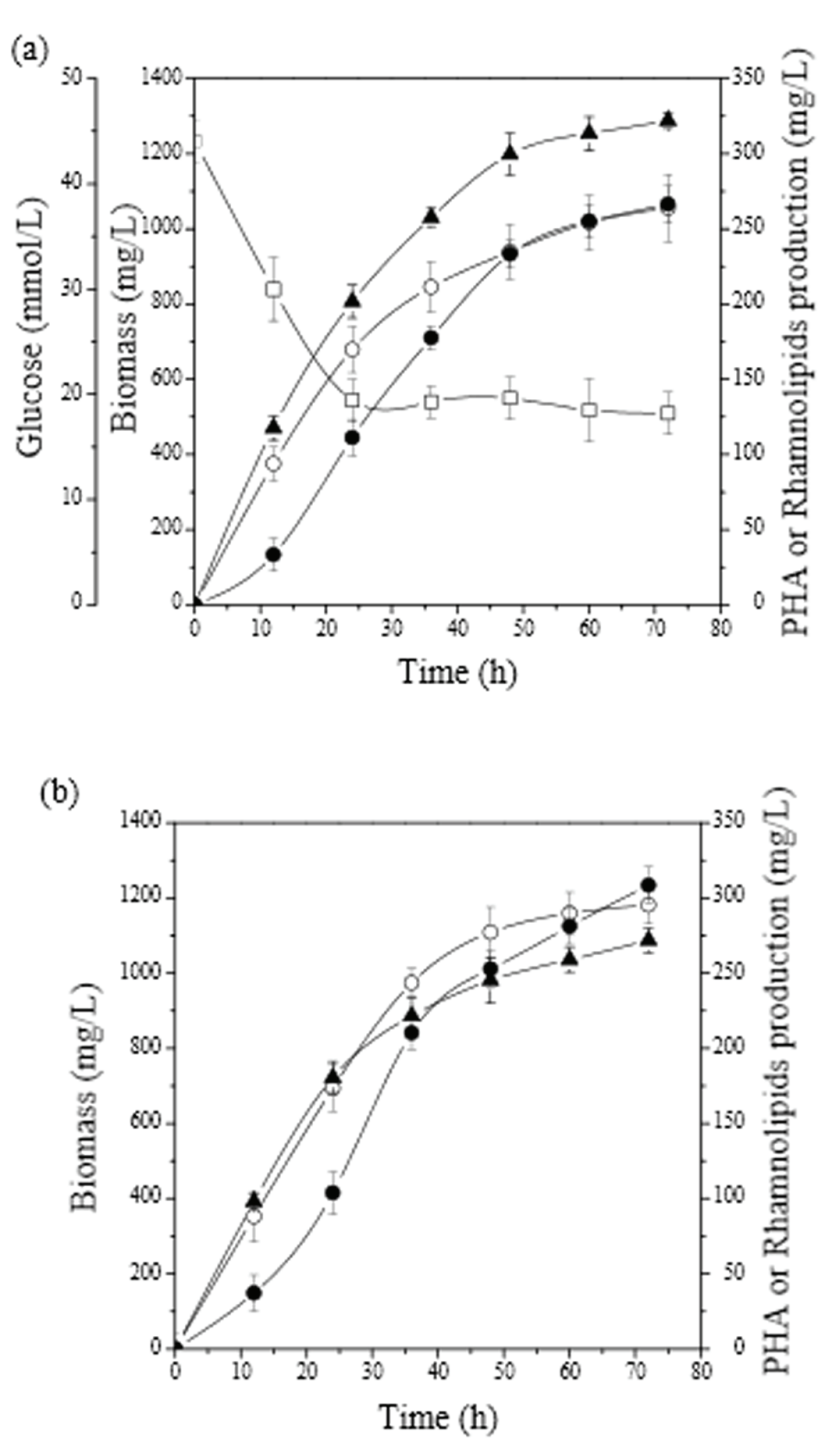
**Simultaneous PHAs and RLs production by *T. thermophilus *HB8 in MSM containing glucose (a) or sodium gluconate (b) as sole carbon source**. Symbols: biomass (closed triangle); PHAs production (closed circles); RLs production (open circles) and glucose concentration (open squares).

Each of the tested carbon sources assured the simultaneous production of PHAs and RLs, whereas biomass was increasing during cultivation as illustrated in Figure 1. Similarly, PHAs and RLs productions in both cultures increased during cultivation process reaching their maximum values at the end of the culture (after 72 h).

The utilization of glucose by *T. thermophilus *resulted in a high biomass production reaching at 1288 mg/L as shown in Figure 1a, while at the same time PHAs and RLs production were 266 mg/L and 189 mg/L respectively. A sharp decrease of glucose concentration was recorded until 20 h of growth; while at prolongated cultivation glucose remained equal to 20 mmole/L until 72 h. However, the value of exhausted glucose that was two thirds of the initial value still remained stable because of the conversion of glucose into acidic compounds such as gluconic acid. It is well known that the cultivation of certain kinds of bacteria on glucose decreases the pH value of the culture broth due to acid production ending in acidification of the culture broth, which might inhibit bacterial growth and might affect also the increase of the PHAs content (Hori et al. 2002).

On the other hand, although the supplementation of MSM with sodium gluconate led to a lower biomass production (1087 mg/L), both PHAs and RLs production were higher (308 mg/L and 211 mg/L respectively) (Figure 1b) than that obtained when glucose was used as sole carbon source. Thus, sodium gluconate was chosen to be tested for the simultaneous PHAs and RLs production in the experiment for optimization of phosphate concentration.

In addition, polymer composition was found to be affected by the carbon sources used. More specific, glucose consumption led to the production of a copolymer containing as monomers 3HD (60.2%), 3HO (20.8 mol%), 3HB (10.5 mol%), and 3HDD (8.5 mol%). When sodium gluconate was utilized as sole carbon source, a copolymer containing both scl and mcl HAs was also accumulated. This copolymer was consisted of 3HD (65 mol%), 3HO (23 mol%) as majors constituents and 3HB (4 mol%) and 3HV (8 mol%) as minor monomer units.

### Effect of initial phosphate concentration [PO_4_^3-^] on simultaneous PHAs and RLs production by *T. thermophilus*

Phosphate is a vital component of many cell structures including nucleic acids, phospholipids, proteins and coenzymes. Phosphate compounds transformations are involved in cellular energetic, and the direction of many biochemical processes in microorganisms is strongly dependent on the presence of phosphates (Dawes 1986; Kulaev 1985). Thus, following the preliminary experiments in which we investigated the influence of carbon source on PHAs and PLs production in culture media containing initially 36 mM of phosphates we examine the influence of various initial phosphate concentrations (ranging from 0.5-50 mM [PO_4_^3-^] on both products synthesis. Sodium gluconate was chosen as sole carbon source since it leads to the higher PHAs and RLs production.

The results revealed that all initial [PO_4_^3-^] tested sustained the growth of *T. thermophilus*, and PHAs and PLs synthesis as well, while in all cultures the values of biomass, PHAs and RLs production recorded after 72 h of cultivation exhibited to have still an increasing tendency. In addition, Table 1 summarizes the results of cell growth (measured as CDW), PHAs and RLs production, PHAs and RLs yields (as percentage of CDW) and PHAs composition at this time of cultivation.

**Table 1:** Effect of the initial phosphate concentration in *T. thermophilus *growth, rhamnolipids production and PHAs production and composition^a^

Phosphate concentration (mM)	Biomass (CDW^b^) (mg/L)	Rhamnolipids (mg/L)	Yield of Rhamnolipids (% DCW)	PHA (mg/L)	PHA content (% DCW)	monomer composition (%mole)			
						
						HB (C_4_)	HV (C_5_)	HO (C_8_)	HD (C_10_)
0.5	667	230	34.48	164	24.28	4.4	8.5	26.7	60.4
5	840	204	24.29	258	30.73	4.6	7.9	26.2	61.3
10	911	202	22.17	298	32.70	4.2	8.8	25.9	61.1
25	1040	161	15.48	362	34.81	5.3	8.1	26.8	59.8
50	1120	118	10.54	228	20.36	4.9	8.3	25.4	61.1

^a ^Measures were obtained after 72 h of cultivation.

^b ^CDW: Cell Dry Weight

Note: all experiments were performed twice while all data are means of triplicates determinations.

**Figure 2:**
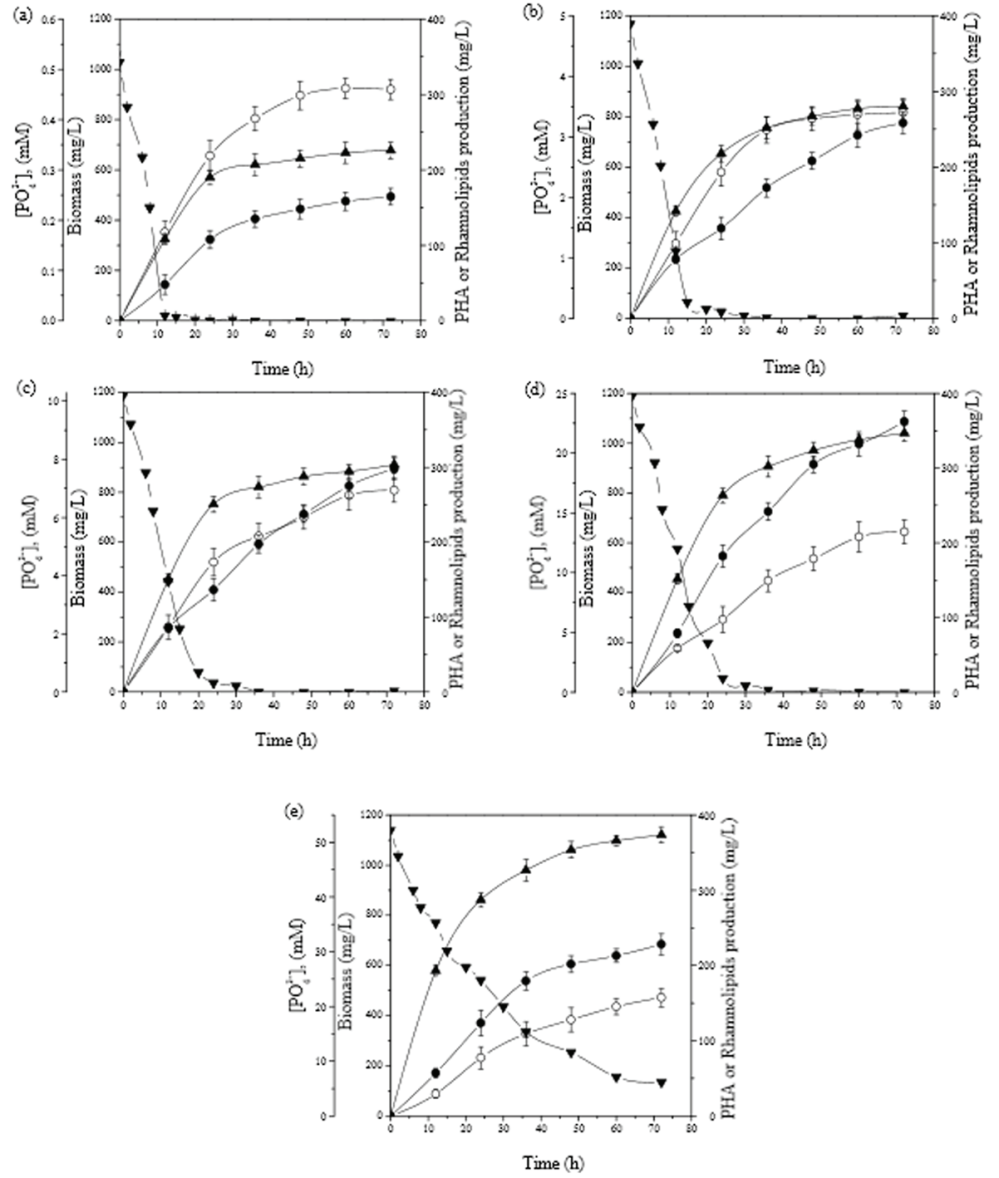
**Effect of the initial [PO_4_^3-^] on *T. thermophilus *HB8 growth, PHAs accumulation and RLs synthesis**. *T. thermophilus *was grown in MSM containing sodium gluconate (1.5% w/v) as sole carbon source and one of the following initial [PO_4_^3-^] 0.5 mM (a); 5 mM (b); 10 mM (c); 25 mM (d) and 50 mM (e). Symbols: Biomass (CDW) (closed triangles); PHAs production (closed circles); RLs (open circles) and [PO_4_^3-^] (reversed closed triangles).

Phosphorus was limited after 10 h of cultivation in the culture medium containing 0.5 mM of initial [PO_4_^3-^] (Figure 2a), resulting in both the lowest cell and PHAs concentration (667 mg/L and 164 mg/L, respectively) recorded in this experiment (Table 1). In addition, PHAs content was only 24% of CDW. In contrast, phosphate limitation resulted in the highest RLs production and yield, reaching 230 mg/L and 34.48% respectively, as illustrated in Table 1. Phosphorus was early limited, approximately after 15 h of cultivation, in the culture medium containing initially 5 mM of [PO_4_^3-^] resulting in a biomass production of 840 mg/L (Figure 2b). PHAs production was increased with the increase of cultivation time reaching a maximum value of 258 mg/L after 72 h of cultivation. On the other hand, RLs production reached a plateau after 30 h of cultivation and a maximal value of 204 mg/L (Figure 2b).

Amongst the cultures tested, biomass production was significant higher, in the culture medium containing 10 mM of [PO_4_^3-^], which were exhausted approximately after 20 h of cultivation (Figure 2c). Both PHAs and RLs production increased during cultivation reaching at 298 mg/L and 202 mg/L, respectively at the end of the culture (72 h). PHAs and RLs yields (as % of CDW) were 22.17% and 32.70%, respectively (Table 1). In the culture containing 25 mM of initial [PO_4_^3-^], cell concentration reached the value of 1040 mg/L after 72 h of cultivation (Figure 2d). Phosphate limitation was achieved after 24 h of cultivation (at the beginning of the stationary phase) resulting in high PHAs production. When phosphate limitation occurred, the rate of cell growth was decreased while PHAs concentration increased rapidly from 110 to 362 mg/L. In contrast, RLs production as well as its yield (% of CDW) were significant lower (161 mg/L and 15.5%, respectively) compared to that recorded in culture medium containing 0.5 mM of initial [PO_4_^3-^].

In contrast, phosphate was not limited in culture media containing initially 50 mM of [PO_4_^3-^], even after 72 h of cultivation, resulting in the highest cell concentration, 1120 mg/L as illustrated in Figure 2e. However, PHAs accumulation was almost 1.3 and 1.5 times respectively lower (228 mg/L) compared to the PHAs production recorded in culture medium containing 10 or 25 mM of phosphate. RLs production was increasing almost linearly during growth reaching at 118 mg/L after 72 h of cultivation. However, RLs production and its yield were 2 and 3 times respectively lower than that obtained in culture medium containing initially 0.5 mM of [PO_4_^3-^] (Table 1). Moreover, the initial [PO_4_^3-^] did not significantly influence the polymer composition as illustrated in Table 1.

### Dependence of simultaneous PHAs and RLs production from cultivation time and initial phosphate concentration [PO_4_^3-^]

**Figure 3:**
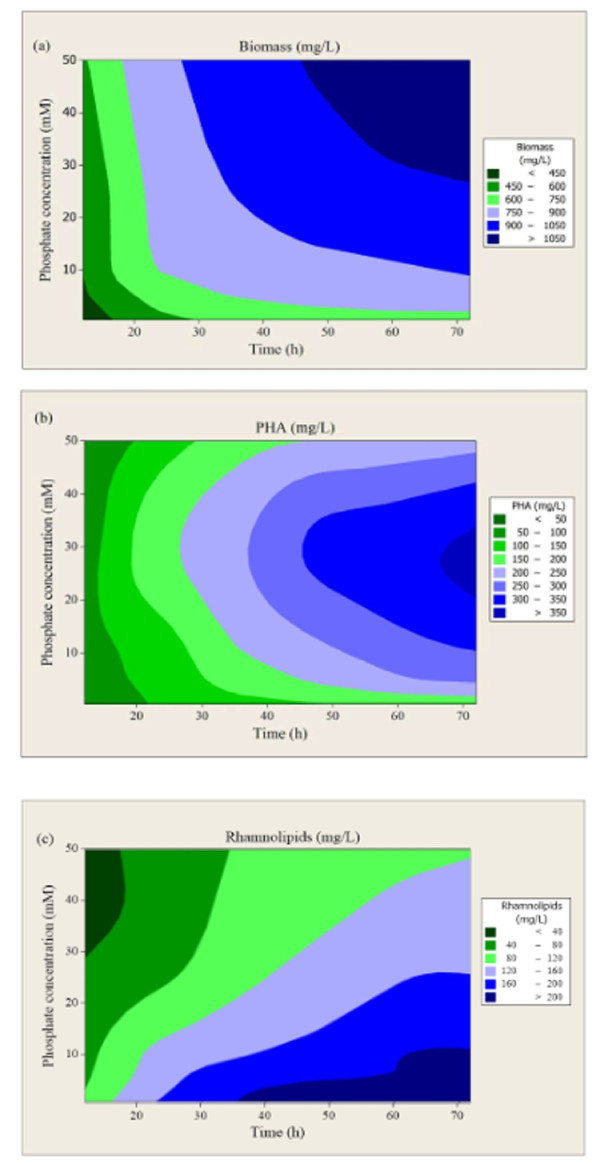
**Contour plots of the combine effect of the initial [PO_4_^3-^] and cultivation time on biomass production (a), PHAs accumulation (b) and RLs synthesis (c) by *T. thermophilus *HB8**.

For the simultaneous PHAs and RLs production, the combine effect of both the cultivation time and the initial [PO_4_^3-^] on biomass was examined, and we designed contour plots using the Minitab V15 software. Figure 2a illustrates the combine effect of both the cultivation time and the initial [PO_4_^3-^] on biomass production. As it can be seen, biomass increased with both the augmentation of the initial [PO_4_^3-^] and the cultivation time. All contours, which represent regions of biomass amplitude (defined in the inset of Figure 3a) exhibited a tendency of convergence at a concrete point that corresponds in the intersection of 50 mM of [PO_4_^3-^] and 72 h of cultivation time. These results indicated that whether the upper [PO_4_^3-^] would have increased more, then the contours would have a convergence at a point. Thus, by increasing further the [PO_4_^3-^] up to 50 mM, biomass seems to come on at a higher value after a more prolonged time of cultivation.

Similarly, the combine effect of both the initial [PO_4_^3-^] and the cultivation time on PHAs production is shown in Figure 3b. In this graph, all contours represent regions of PHAs production amplitude (defined in the inset of Figure 3b). In these contours, PHAs production increased (> 300 mg/L) with the increase of cultivation time but the convergence of the curves toward the initial phosphates concentrations of 25-30 mM demonstrated that PHAs production would not be further rise by increasing phosphates concentration above 30 mM. This value corresponds to the intersection of the lines at the 30 mM of phosphates concentration and at 72 h of cultivation time.

Furthermore, the influence of both the initial phosphates concentration and the cultivation time on rhamnolipids synthesis is illustrated in Figure 3c. In this graph, all contours represent regions of rhamnolipids production amplitude (defined in the inset of Figure 3c). As it can be seen rhamnolipids synthesis occurred after 35 h of cultivation and continued until the 72 h of cultivation, where the [PO_4_^3-^] is lower than 10 mM. RLs concentration increased (> 200 mg/L) with the increase of cultivation time and the simultaneous decrease of the initial phosphate concentration, while high [PO_4_^3-^] had a negative effect on RLs production.

### TLC results

Biochemical analysis of RLs biosynthesis is confirmed by TLC. The wild type strain of *T. thermophilus *HB8 make all RL biosynthetic intermediates under the described growth conditions and produces two α-thymol positive spots-mainly the slow-migrating spot, which represents di-RLs (Rf 0.125) and secondly the fast-migrating spot representing mono-RLs (Rf 0.56), in the presence of sodium gluconate. It is queer that in the presence of glucose only di-RLs was detected. This result is probably due to a quantitative difference of sample or a degradation of di-RLs to mono-RLs in the case of sodium gluconate. Ramnose and saponin white were used as sample of reference (data not shown).

### LC-MS characterization

The mixture of RLs was also analyzed by direct infusion of the sample into the mass spectrometer. As described in the Experimental Section, the MS spectra were acquired in both positive and negative ion modes to confirm the structural assignment of the quasi-molecular ion, [M + H]^+ ^in positive ion mode by the corresponding molecular anion [M - H]^-^. Assignments of the mass spectra ions were aided by elemental composition calculation results, comparison of structural analogues and available literature (Camilios-Neto et al. 2010; Camilios Neto et al. 2009; Déziel et al. 1999; Haba et al. 2003; Lotfabad et al. 2010; Monteiro et al. 2007; Pantazaki et al. 2010; Price et al. 2009; Rooney et al. 2009) as well as acquired knowledge regarding mass spectrometry of related compounds. Table 2 depicts the average spectrum of a RLs-containing sample at 72 h, which was obtained using the infusion method in the positive mode. In most cases, the positive spectra exhibited an [M+H]^+ ^pseudomolecular ion, which was accompanied by a series of adduct ions (i.e. [M + Na]^+^, [M+K]^+^, [M-H+Na_2_]^+^, [M+H+CH_3_]+ etc.) due to the presence of methanol, water and formic acid into the sample. The spectra were characterized by molecular ions for mono-RLs in the mass range from 303-673, di-rhamno-mono-lipidic congeners from 453-509 and di-RLs from 595 to 792 mass units, respectively. The protonated ions [M+H]^+ ^at m/z 447, m/z 587, and m/z 645 correspond to mono-RLs Rha-C_8_-C_8:1_/Rha-C_8:1_-C_8_/Rha-C_12_-C_14:1_/Rha-C_14_-C_12:1 _and Rha-C_14_-C_16_/Rha-C_16_-C_14_, respectively. The ions at m/z 621, m/z 703, m/z 733 and m/z 792, correspond to di-RLs Rha_2_-C_8_-C_10:1_/Rha_2_-C_8:1_-C_10_/Rha_2_-C_8:1_-C_10_/Rha_2_-C_10:1_-C_8_, Rha_2_-C_12_-C_12:2_/Rha_2_-C_12:2_-C_12_, Rha_2_-C_12_-C_14:1_/Rha_2_-C_14:1_-C_12_/Rha_2_-C_14_-C_12:1_/Rha_2_-C_12:1_-C_14_, and Rha_2_-C_16_-C_14_/Rha_2_-C_14_-C_16 _that were the most predominant ones. Major ions at m/z 471 and m/z 553 are attributed to the [M+Na]+ adduct ions for the two major mono-RLs, Rha-C_8_-C_8 _and Rha-C_10_-C_12:1_/Rha-C_10:1_-C_12_, respectively and ions at m/z 645, m/z 671, and m/z 673 are attributed to the [M+Na]^+ ^adduct ions for the three major di-RLs, Rha_2_-C_10_-C_8_/Rha_2_-C_8_-C_10_, Rha_2_-C_10_-C_10:1_/Rha_2_-C_10:1_-C_10 _and Rha_2_-C_10_-C_10_, respectively. Other characteristic ions in mass spectrum appeared at m/z 661 and m/z 689, which corresponds to the molecular [M+K]^+ ^potassium adduct ions of di-RLs, Rha_2_-C_10_-C_8_/Rha_2_-C_8_-C_10 _and Rha_2_-C_10_-C_10_, respectively. The molecular [M-H+Na_2_]^+ ^di-sodium adduct ions at m/z 667 and 695 were also observed for these RLs but in much lower abundance. Moreover, major [M-H+Na_2_]^+ ^molecular ion at m/z 747 is assigned as Rha_2_-C_12_-C_12:2_/Rha_2_-C_12:2_-C_12_. Additionally, the [M+Na+14]+ ions at m/z 517 and at m/z 663 are due to the methyl esters of mono-RLs with some unsaturated fatty acids [Rha-(C_10_-C_10:1_)]/[Rha-(C_10:1_-C_10_)] and di-RLs Rha_2_-C_10_-C_10:1_/Rha_2_-C_10:1_-C_10_, respectively. Ions assignments are in accordance with the earlier work (Price et al. 2009).

**Table 2:** Identification and Characterization of major Rhamnolipids compounds using LC-ESI-MS (Both in negative and positive mode).

Substance	[M+H]^+ ^*m*/*z*	[M+Na]^+ ^*m*/*z*	[M+K]^+ ^*m*/*z*	[M-H+Na_2_]^+ ^*m*/*z*	[M-H]^- ^*m*/*z*
**Mono-rhamno-mono-lipidic congeners**					
[Rha-C_8:2_]	303		341		301
[Rha-C_8:1_]	305				
[Rha-C_10:2_]	331				
[Rha-C_10:1_]	333				331
[Rha-C_10_]	335				
[Rha-C_12:2_]	359	381			
Octyl phathalate		**413**			
**Mono-rhamno-di-lipidic congeners**					
[Rha-(C_8_-C_8:1_)] or [Rha-(C_8:1_-C_8_)]	**447**		**485**	**491**	445
[Rha-(C_8_-C_8_)]	449	**471**	487	**493**	447
[Rha-(C_10_-C_8_)] or [Rha-(C_8_-C_10_)]	477	499	**515**	521	475
[Rha-(C_8_-C_10:1_)] or [Rha-(C_8:1_-C_10_)]	475				473
[Rha-(C_10_-C_10:1_)] or [Rha-(C_10:1_-C_10_)]	**503**	525	**541**	**547**	501
[Rha-(C_10_-C_10_)]	**505**		**543**		503
[Rha-(C_10_-C_10:1_)-CH_3_] or [Rha-(C_10:1_-C_10_)-CH_3_]	**517**				
[Rha-(C_10_-C_12:1_)] or [Rha-(C_10:1_-C_12_)]	**531**	**553**	569	575	**529**
[Rha-(C_10_-C_12_)] or [Rha-(C_12_-C_10_)]	**533**	555	**571**	577	**531**
[Rha-(C_12_-C_14:1_)] or [Rha-(C_14_-C_12:1_)]	**587**		**625**	**631**	585
[Rha-(C_14_-C_14_)]	**617**			**661**	
[Rha-(C_14_-C_14:1_)] or [Rha-(C_14:1_-C_14_)]	615				**613**
[Rha-(C_14_-C_16_)] or [Rha-(C_16_-C_14_)]	**645**				643
[Rha-(C_14_-C_16:1_)] or [Rha-(C_14_-C_16:1_)]	643				**641**
[Rha-(C_16_-C_16_)]	673				**671**
**Di-rhamno-mono-lipidic congeners**					
[Rha-Rha-C_8_)]	453				451
[Rha-Rha-C_10:1_)]	**479**				**477**
[Rha-Rha-C_10_)]	**481**				**479**
[Rha-Rha-C_12:1_)]	**507**				505
[Rha-Rha-C_12_)]	509				**507**
**Di-rhamno-di-lipidic congeners**					
[Rha-Rha-(C_8_-C_8_)]	**595**	**617**	633		593
[Rha-Rha-(C_8_-C_10:1_)] or [Rha-Rha-(C_8:1_-C_10_)] [Rha-Rha-(C_10_-C_8:1_)] or [Rha-Rha-(C_10:1_-C_8_)]	**621**		**659**	**665**	**619**
[Rha-Rha-(C_10_-C_8_)] or [Rha-Rha-(C_8_-C_10_)]	623	**645**	**661**	667	621
[Rha-Rha-(C_10_-C_10_)]	651	**673**	**689**	695	649
[Rha-Rha-(C_10_-C_10:1_)] or [Rha-Rha-(C_10:1_-C_10_)] [Rha-Rha-(C_8_-C_12:1_)] or [Rha-Rha-(C_8:1_-C_12_)]	649	**671**	687	693	**647**
[Rha-Rha-(C_10_-C_10:1_)-CH_3_] or [Rha-Rha-(C_10:1_-C_10_)-CH_3_]	**663**				
[Rha-Rha-(C_10_-C_12:1_)] or [Rha-Rha-(C_10:1_-C_12_)] [Rha-Rha-(C_12:1_-C_10_)] or [Rha-Rha-(C_12_-C_10:1_)]	677	**699**	715	**721**	675
[Rha-Rha-(C_10_-C_12_)] or [Rha-Rha-(C_12_-C_10_)]	**679**	**701**	717	723	677
[Rha-Rha-(C_12_-C_12:1_)] or [Rha-Rha-(C_12:1_-C_12_)]	**705**	727	743	**749**	703
[Rha-Rha-(C_12_-C_12:2_)] or [Rha-Rha-(C_12:2_-C_12_)]	**703**	725	741	**747**	701
[Rha-Rha-(C_12_-C_12_)]	707	729		751	705
[Rha-Rha-(C_12_-C_14:1_)] or [Rha-Rha-(C_14:1_-C_12_)] [Rha-Rha-(C_14_-C_12:1_)] or [Rha-Rha-(C_12:1_-C_14_)]	**733**			**777**	731
[Rha-Rha-(C_14_-C_14:1_)] or [Rha-Rha-(C_14:1_-C_14_)]	**762**	784		806	760
[Rha-Rha-(C_16_-C_14_)] or [Rha-Rha-(C_14_-C_16_)]	**792**	**814**			**790**

Data shown in bold represent major components of the corresponding Rhamnolipid mixture

Similarly to the positive ionization, mono-RLs Rha-C_10_-C_12_/Rha-C_12_-C_10 _(m/z 531), Rha-C_10_-C_10 _(m/z 503), Rha-C_8_-C_10_/Rha-C_8_-C_10 _(m/z 475) and di-RLs Rha_2_-C_10_-C_12_/Rha_2_-C_12_-C_10 _(m/z 677), Rha_2_-C_12_-C_12 _(m/z 705), and Rha_2_-C_10_-C_10 _(m/z 649), have been revealed as the main components in the RLs mixture by negative ESI (Table 2). Also, four components with unsaturated fatty acids were found among RLs homologues, which were detected as the anions of m/z 529, 647, 675 and 703. These correspond to the de-protonated molecules of RLs, Rha-C_10_-C_12:1_/Rha-C_10:1_-C_12_, Rha_2_-C_10_-C_10:1_/Rha_2_-C_10:1_-C_10_/Rha_2_-C_8_-C_12:1_/Rha_2_-C_8:1_-C_12_, Rha_2_-C_10_-C_12:1_/Rha_2_-(C_10:1_-C_12_/Rha_2_-C_12:1_-C_10_/Rha_2_-C_12_-C_10:1 _and Rha_2_-C_12_-C_12:1_/Rha_2_-C_12:1_-C_12_, respectively. Furthermore, some fragments in the mass range 163 - 503 mass arising from the carbohydrate moieties were also evident in the mass spectra, appearing as de-protonated species. Most of the ions above m/z 447 are RLs pseudo-molecular [M - H]- ions and the most ions between m/z 163 and 503 are fragment ions produced by cleavage (Déziel et al. 1999). The characteristic ion in mass spectrum appeared at m/z 311, corresponds to the rupture of the link in the rhamnose-alkylic chain of di-RLs. The MS spectrum reveals also a key fragment ion at m/z 293, which comes from a loss of water molecule. Characteristic ion at m/z 339 corresponding to removal of an entire rhamnose unit (m/z 163) while m/z 333 indicating removal of a C_10 _fatty acid chain from a mono-RL Rha-C_20 _(m/z 503) were also present. These results clearly show that Rha-C_10_-C_10 _RL homolog is the predominant one compared to a mixture of Rha-C_8_-C_12 _and Rha-C_12_-C_8 _(Camilios Neto et al. 2009). Major ions at m/z 283, m/z 393 and m/z 421 are attributed to removal of an entire rhamnose unit from Rha-C_8_-C_8 _(m/z 163), and an di-rhamnose unit (m/z 309), from RLs with some unsaturated fatty acid, Rha_2_-C_12_-C_12:1_/Rha_2_-C_12:1_-C_12 _and Rha_2_-C_12_-C_14:1_/Rha_2_-C_14:1_-C_12_/Rha_2_-C_14_-C_12:1_/Rha_2_-C_12:1_-C_14_, respectively. The observed 28 mass unit difference between m/z 393 and m/z 421 ions corresponds to the mass difference between the C_12 _and C_14 _acyl moiety. Characteristic ions of rhamnose and di-rhamnose moiety were also appeared at m/z 163 and 309 with low relative abundance. Another characteristic ion at m/z 247 that might potentially have been produced by the fragmentation of Rha_2_-C_10_-C_10 _(Camilios-Neto et al. 2010; Zgola-Grzéskowiak and Kaczorek 2011) and Rha_2_-C_10_-C_12 _and Rha_2_-C_10_-C_12:1 _(Monteiro et al. 2007) RLs was also present. The available information provided by mass spectrometry was not sufficient to precisely elucidate the structure of this ion. The characterization of negative ESI MS spectra is consistent with earlier studies (Camilios-Neto et al. 2010; Monteiro et al. 2007). By comparing the two ionization modes, it appears that both were sensitive and suitable for the majority of the analyzed compounds. Each mode provided complementary information that enabled the full and unambiguous identification of the RLs homologues.

## Discussion

*T. thermophilus *was demonstrated to be capable to produce simultaneously two biotechnologically important bio-products; biodegradable biopolymers PHAs and RLs-type biosurfactants by utilizing either glucose or sodium gluconate as sole carbon source, although the ability to produce them, and especially RLs, is in fact restricted to a limited number of bacterial species (Abdel-Mawgoud et al. 2010). One of the long-term interests of the scientists is to produce RLs in strains other than the opportunistic known pathogen *P. aeruginosa *(Chwalek et al. 2006) due to the wide field of their applications. This paper contributes to this goal taken into account the advantages of *T. thermophilus *HB8 due to the exceptionality of the non-pathogenic and thermophilic nature of this strain, and its capability of naturally producing these metabolites. Apart from the advantages resulting from the thermophilicity of the microorganism that reflects positively on the solubility of substrates and the reduced likelihood of contamination should be taken into account the requirement of high temperature of 75°C and consequently the high cost of energy for bacterial growth, and production of both bio-products. A good compensation for overcoming this problem will be the use of inexpensive wastes substrates.

The usage of *T. thermophilus *for their production in low cost seems highly promising due to the high solubility of suitable carbon sources located in wastes, especially of water-immiscible substrates, where RLs production was high. The consumption of gluconate as sole carbon source by *T. thermophilus *resulted in higher production of both metabolites than that when glucose was used. Among the various initial [PO_4_^3-^] tested, the medium containing initially 25 mM of [PO_4_^3-^] resulted in the higher polymer production after 72 h of cultivation (Table 1), consistent with the report that the initial [PO_4_^3-^] in the culture medium influences both the cell density and the PHAs production (Lee et al. 2000). The recovery of PHAs requires the disruption of valuable biocatalysts of bacterial cells, while the production of secreted materials, such as RLs, in combination with PHAs production is expected to enhance the availability of biocatalysts because the cells can be used as "microbial cell factories" for the production of the exo-products before the eventual disruption to recover PHAs.

The investigation of the combine effect of both the cultivation time and the initial [PO_4_^3-^] on biomass, and on simultaneous PHAs and RLs production was evaluated from contour plots (Figure 3). As it was revealed, biomass was increased by increasing both initial [PO_4_^3-^] and cultivation time in the experimental ranges tested. However, a further increase of the initial [PO_4_^3-^] and cultivation time would lead the contours to convergence at a concrete point (Figure 3a). Moreover, PHAs production was increasing during cultivation time but there would not be further rise in PHAs production obtained by increasing [PO_4_^3-^] above a crucial concentration, i.e. above 30 mM (Figure 3b), as it can be deduced by the convergence of the curve toward the center point of initial phosphate concentration. In contrast, the increase of the initial [PO_4_^3-^] had a negative effect on RLs production, but the biosurfactant concentration increased during cultivation time (Figure 3c). These results revealed that PHAs synthesis occurred only during active cell growth, while substantial RLs production began early but is completed at the onset of the stationary phase. This strategy might be useful as the beginning to optimize other parameters affecting these processes such as nitrogen, carbon sources etc.

PHAs synthesized in both carbon sources are copolymers with a very similar composition of monomers possessing carbon chain lengths ranging from 6-12 carbon atoms. Since PHAs and RLs syntheses have a close metabolic relation, since (R)-3-hydroxyfatty acids are used as common precursors in both pathways, it has been proposed that both PHAs and RLs biosynthesis pathways may compete for (R)-3-hydroxyfatty acid ACP (Rehm et al. 2001; Soberón-Chávez et al. 2005). However, there is still no clear evidence for this probable competitive relation. A regulatory switch of the flow of beta-hydroxyalkanoic acids from PHAs polymerization to RLs synthesis is clearly indicated to occur when the culture reaches the stationary phase.

Recently, a proposed distributional pathways of (R)-3-hydroxyacyl groups for the synthesis of PHAs, HAAs ((R)-3-((R)-3-hydroxyalkanoyloxy) alkanoic acids), mono-RLs and di-RLs in *Pseudomonas aeruginosa *grown on medium-chain-length fatty acids was published. It was suggested that during the exponential cell growth, cells excessively produced free acids 3HAs (such as 3HO, 3HD, etc.), which are secreted into medium and retake-up later for RLs syntheses (Choi et al. 2011). An interrelationship between PHAs and RLs synthesis was found that in rhamnolipid-negative mutants, more (R)-3-hydroxyfatty acid precursors were fluxed toward PHA synthesis. However, the production of rhamnolipid in *P. aeruginosa *seemed to be very tightly regulated at the transcriptional level by the quorum-sensing response and by environmental conditions. Finally, investigation of metabolic pathway for PHAs and RLs synthesis using octanoic-1-^13^C acid suggested that the fatty acid substrate was converted to PHAs and the acyl groups in the RLs with a minimum number of rearrangements (Choi et al. 2011).

Concerning *T. thermophilus *HB8, there is no other information apart of our works for the biosynthetic pathway of PHAs and RLs neither for the corresponding genes in the genome, except that only a rhamnosyl-transferase (YP-144151; TTHA00885) was found, exhibiting a calculated molecular weight of 32,783, and consisting of 290 amino acids (Pantazaki et al. 2010). Recently it was also demonstrated the potential production of RLs by fatty acids by cultivating this bacterium in the presence of sunflower seed oil or oleic acid as carbon sources (Pantazaki et al. 2010) or by sugars such as glucose and its derivative sodium gluconate in this work. Taking into account that *T. thermophilus *produce mcl-PHAs, in which 3-hydroxydecanoate constituted the major constituent when grown in the presence of gluconate, and since 3-hydroxydecanoate can be formed via β-oxidation or via fatty acid de novo biosynthesis, we can suggest that both biosynthesis pathways, might be involved in RLs biosynthesis in this bacterium dependent on the carbon source used (Pantazaki et al. 2010). The β-oxidation leads to the desired R-configurated hydroxy acid and the de novo biosynthesis at first to the S-configurated compound (additional biochemical reactions are necessary to give also in this case the accurate configuration of the polymer precursor). These results confirmed that the type of RLs produced depends on multiple conditions, including the bacterial strain and the carbon source used as previously reported (Mulligan and Gibbs 1993; Robert et al. 1989).

The extracted mixture of RLs produced in the presence of sodium gluconate was subjected to identification and characterization using LC-(ESI)-MS. LC-MS analyses revealed that both mono- and di-RL moieties as well as di-rhamno-mono-lipidic congeners were present in the RLs mixture. Additionally, a plethora of diversity of mono- and di-RLs as well, was identified differing in the length of the lipidic chain, which additionally found to be saturated or unsaturated in some cases (Table 2). The combination of negative and positive ESI modes has been shown to be powerful tools in the characterization of RLs homologues. The wild type strain of *T. thermophilus *HB8 make all RL biosynthetic intermediates under the described growth conditions. This approach could be further applied successfully for the structural elucidation of biosurfactants producing from the substrate mixture of others sources of carbon.

The detailed and extensive RLs characterization of this work demonstrated that a plethora of diversity of mono- and di-RLs were identified differing in the length of the lipidic chain, which additionally found to be saturated or unsaturated in some cases. However, the diversity of β-hydroxyalkanoic acids that are participated in the PHAs synthesized by *T. thermophilus *was not as wide as this of β-hydroxyalkanoic acids that was involved in RLs synthesis and not at all so similar. Moreover, it is known that the composition of PHAs depends on substrate specificity of the PHA polymerases which uses (R)-3-hydroxyalkanoyl-CoA as substrates (Lee et al. 2004). This observation directs to the bold suggestion that RLs production might constitutes a mechanism of exhaustion of the accumulated β-hydroxyalkanoic acids unused "wastes" by-products from the cells to the exterior medium, since in this bacterium anyhow the RLs production began early but is completed at the onset of the stationary phase.

Secreted RLs are recorded as virulence factors of the producing microorganisms (Abdel-Mawgoud et al. 2010). Among the RLs constituents, there is evidence that attributes the implication of the lipidic chain to triggering of some biological effects such as cytotoxicity or heamolytic activity. RLs possessing the same glycoside chain can also have a different influence when the aglycone is only slightly modified. The hydrophobic aglycone backbone could probably intercalate into the hydrophobic membrane bi-layer, while the glycoside chain could interact with the polar group (Chwalek et al. 2006).

Surface-active compounds commonly used by industry are chemically synthesized, and their replacement with bio-surfactants could provide advantages. Bio-surfactants compared to chemically synthesized surfactants have been receiving increasing attention as a result of their unique properties, mild production conditions, lower environmental toxicity, and higher biodegradability (Mulligan 2005; Rosenberg and Ron 1999). However, the use of bio-surfactants has been limited currently due to their relatively high production cost since the main factor limiting commercialization of bio-surfactants is associated with non-economical large scale production. To surmount this obstacle and to compete with synthetic surfactants, inexpensive substrates and effective microorganisms must be developed for bio-surfactant production. Agro-industrial wastes are considered as the most promising substrates for low-cost bio-surfactant production and can alleviate many processing industrial waste management problems. This would lead to the greater possibility for economical bio-surfactant production and reduced pollution caused by those wastes (Maneerat 2005). Aforementioned, many microorganisms, especially *Pseudomonads*, were reported use various low-cost renewable resources as potential carbon sources, but they cannot produce bio-surfactants or only have very low yield. Screening of high RLs-producing microorganisms from the natural environment is a good choice, for RLs production.

These efficient cultivation processes and their mechanisms as they pertain to the simultaneous production of PHAs and RLs encourage us to the use of a range of inexpensive carbon sources containing sugars or fatty acids. The establishment of lab-scale bioreactor experiments that will design in a future project that will include more factors affecting both biosynthetic processes might significantly increase the production of both bio-products, PHAs and RLs.

## Competing interests

The author declares that they have no competing interests.
